# Poly[1,4-bis­(ammonio­meth­yl)cyclo­hexane [di-μ-chlorido-dichloridoplumbate(II)]]

**DOI:** 10.1107/S1600536810016818

**Published:** 2010-05-15

**Authors:** Matthew Kyle Rayner, David Gordon Billing

**Affiliations:** a Molecular Sciences Institute, School of Chemistry, University of the Witwatersrand, Private Bag 3, PO Wits 2050, South Africa

## Abstract

The title compound, {(C_8_H_20_N_2_)[PbCl_4_]}_*n*_, crystallizes as an layered inorganic–organic hybrid perovskite-type structure. Corner-sharing PbCl_6_ octa­hedra extend parallel to the *ac* plane. Adjacent layers are staggered relative to one another, with diammonium cations separating these layers. The cations exhibit 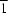 symmetry and inter­act with the inorganic sheets *via* N—H⋯Cl hydrogen bonding in the right-angled halogen sub-type of the terminal halide hydrogen-bonding motif.

## Related literature

Similar structures have been reported by Billing & Lemmerer (2006 ▶) and Dobrzycki & Woźniak (2009 ▶). Structure–properties relation experiments have been performed by Mitzi *et al.* (2001 ▶). For hydrogen-bonding nomenclature for inorganic–organic hybrids, see: Mitzi (1999 ▶). For the bromido- and iodidoplumbate(II) analogues of the title compound, see: Rayner & Billing (2010*a* ▶,*b* ▶).
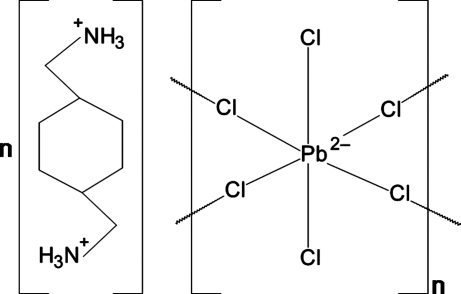


## Experimental

### 

#### Crystal data

(C_8_H_20_N_2_)[PbCl_4_]
*M* *_r_* = 493.25
Orthorhombic, 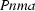

*a* = 7.7990 (2) Å
*b* = 24.0666 (6) Å
*c* = 7.9348 (2) Å
*V* = 1489.33 (7) Å^3^
*Z* = 4
Mo *K*α radiation
μ = 12.02 mm^−1^
*T* = 173 K
0.54 × 0.41 × 0.04 mm

#### Data collection

Bruker APEXII CCD area-detector diffractometer
Absorption correction: integration (*XPREP*; Bruker, 2005 ▶) *T*_min_ = 0.032, *T*_max_ = 0.685
13290 measured reflections
1850 independent reflections
1654 reflections with *I* > 2σ(*I*)
*R*_int_ = 0.049

#### Refinement

*R*[*F* ^2^ > 2σ(*F* ^2^)] = 0.031
*wR*(*F* ^2^) = 0.067
*S* = 1.16
1850 reflections
73 parameters
H-atom parameters constrained
Δρ_max_ = 1.47 e Å^−3^
Δρ_min_ = −3.53 e Å^−3^

### 

Data collection: *APEX2* (Bruker, 2005 ▶); cell refinement: *SAINT* (Bruker, 2005 ▶); data reduction: *SAINT*; program(s) used to solve structure: *SHELXS97* (Sheldrick, 2008 ▶); program(s) used to refine structure: *SHELXL97* (Sheldrick, 2008 ▶); molecular graphics: *ORTEP-3 for Windows* (Farrugia, 1997 ▶) and *DIAMOND* (Brandenburg, 1999 ▶); software used to prepare material for publication: *WinGX* (Farrugia, 1999 ▶) and *PLATON* (Spek, 2009 ▶).

## Supplementary Material

Crystal structure: contains datablocks I, global. DOI: 10.1107/S1600536810016818/wm2339sup1.cif

Structure factors: contains datablocks I. DOI: 10.1107/S1600536810016818/wm2339Isup2.hkl

Additional supplementary materials:  crystallographic information; 3D view; checkCIF report

## Figures and Tables

**Table 1:** Selected bond lengths (Å)

Pb1—Cl3^i^	2.834 (2)
Pb1—Cl1^ii^	2.8723 (15)
Pb1—Cl2^iii^	2.900 (2)

Symmetry codes: (i) 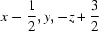; (ii) 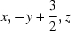; (iii) 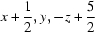.

**Table 2:** Hydrogen-bond geometry (Å, °)

*D*—H⋯*A*	*D*—H	H⋯*A*	*D*⋯*A*	*D*—H⋯*A*
N1—H1*C*⋯Cl3^iv^	0.91	2.40	3.249 (5)	156
N1—H1*D*⋯Cl1^v^	0.91	2.44	3.196 (6)	141
N1—H1*E*⋯Cl1^iii^	0.91	2.39	3.212 (5)	150
N1—H1*E*⋯Cl2^iii^	0.91	2.84	3.337 (5)	115

Symmetry codes: (iii) 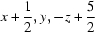; (iv) 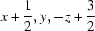; (v) 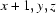.

**Fig. 1.**
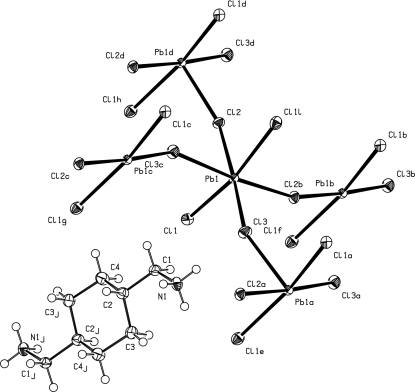
The asymmetric unit of the title compound with atom labels. Displacement ellipsoids were drawn at the 50% probability level. Symmetry codes: (a) -1/2+x, 1/2-y, 3/2-z (b) 1/2+x, 1/2-y, 1/2-z (c) x, 1/2-y, z (d) 1-x, -y, -z.

**Fig. 2.**
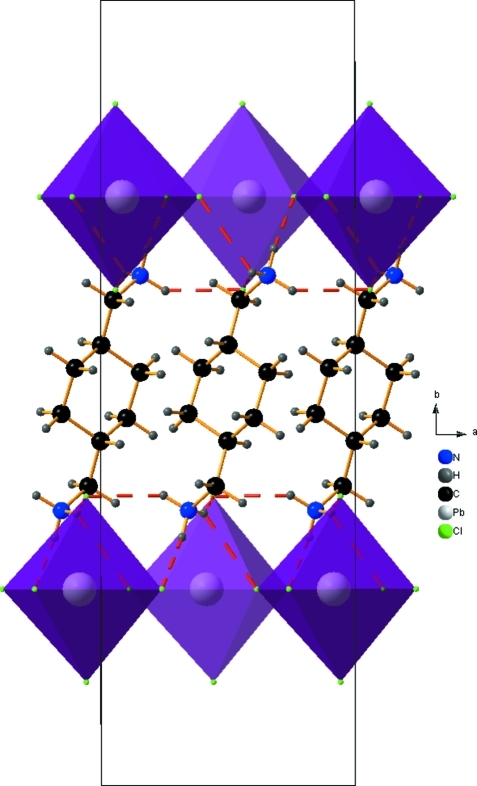
Packing diagram viewed along the b axis. Hydrogen bonds are drawn as dashed red lines.

**Fig. 3.**
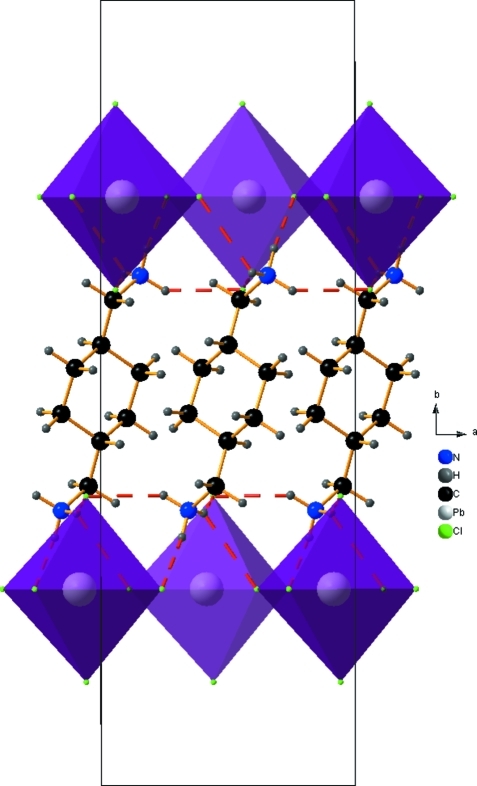
Packing diagram viewed along the c axis. Hydrogen bonds are drawn as dashed red lines.

## supplementary crystallographic information

### Comment

Inorganic-organic hybrid compounds have been investigated for their semiconduting and electronic properties (Mitzi *et al.*, 2001). For literature regarding hydrogen bonding nomenclature for inorganic-organic hybrids, see: Mitzi (1999). The title structure (Fig. 1) is one of three 2-dimensional hybrid structures that we have synthesized encorporating this diammonium cation. The structures differ in terms of their halogen ligands, which include chloride (presented here), bromide (Rayner & Billing, 2010*a*) and iodide (Rayner & Billing, 2010*b*). The bromide and iodide hybrids crystallize in the monoclinic system with space group *P*2_1_/*c* while the chloride hybrid crystallizes in the orthorhombic, *Pnma* system.

In the title structure the lead-chloride octahedra from alternate layers that are staggered relative to one another (Fig. 2). In all three structures only the *trans* form of the cation has been observed, giving the cation 1 symmetry (Fig. 3). The ammonium cations interact with the inorganic layer via N—H···*X* (*X* = Br, I and Cl) hydrogen bonding in the right-angled halogen subtype of the terminal halide hydrogen bonding motif (Mitzi, 1999). Similar inorganic-organic hybrid structures have been reported (Billing & Lemmerer, 2006; Dobrzycki & Woźniak, 2009), however very few hybrids encorporating diammonium cations have been synthesized.

### Experimental

A mixture of 0.052 g (0.19 mmol) PbCl_2_ and 0.030 g (0.21 mmol) 1,4-bis-(aminomethyl)-cyclohexane (mixture of isomers) was dissolved in 5 ml HCl at 383 K and slow cooled at a rate of 0.069 K/min to yield colourless, plate-shaped single crystals suitable for X-ray analysis.

### Refinement

The H atoms on the diammonium cation were refined using a riding-model, with C—H = 0.99 Å, N—H = 0.91 Å and with U_iso_(H)=1.2*U*_eq_(C) or 1.5*U*_eq_(N). The highest residual electron density peak (1.47 e Å-3) was 0.822Å from Pb1.

### Crystal data

**Table:**

(C_8_H_20_N_2_)[PbCl_4_]	*F*(000) = 928
*M**_r_* = 493.25	*D*_x_ = 2.200 Mg m^−^^3^
Orthorhombic, *P**n**m**a*	Mo *K*α radiation, λ = 0.71073 Å
Hall symbol: -P 2ac 2n	Cell parameters from 6650 reflections
*a* = 7.7990 (2) Å	θ = 2.7–28.3°
*b* = 24.0666 (6) Å	µ = 12.02 mm^−^^1^
*c* = 7.9348 (2) Å	*T* = 173 K
*V* = 1489.33 (7) Å^3^	Plate, colourless
*Z* = 4	0.54 × 0.41 × 0.04 mm

### Data collection

**Table:**

Bruker APEXII CCD area-detector diffractometer	1850 independent reflections
Radiation source: fine-focus sealed tube	1654 reflections with *I* > 2σ(*I*)
graphite	*R*_int_ = 0.049
φ and ω scans	θ_max_ = 28.0°, θ_min_ = 1.7°
Absorption correction: integration (*XPREP*; Bruker, 2005)	*h* = −10→10
*T*_min_ = 0.032, *T*_max_ = 0.685	*k* = −31→31
13290 measured reflections	*l* = −10→10

### Refinement

**Table:**

Refinement on *F*^2^	Primary atom site location: structure-invariant direct methods
Least-squares matrix: full	Secondary atom site location: difference Fourier map
*R*[*F*^2^ > 2σ(*F*^2^)] = 0.031	Hydrogen site location: inferred from neighbouring sites
*wR*(*F*^2^) = 0.067	H-atom parameters constrained
*S* = 1.16	*w* = 1/[σ^2^(*F*_o_^2^) + (0.003*P*)^2^ + 19.9694*P*] where *P* = (*F*_o_^2^ + 2*F*_c_^2^)/3
1850 reflections	(Δ/σ)_max_ = 0.007
73 parameters	Δρ_max_ = 1.47 e Å^−^^3^
0 restraints	Δρ_min_ = −3.53 e Å^−^^3^

### Special details

**Table:**

Experimental. Numerical intergration absorption corrections based on indexed crystal faces were applied using the XPREP routine (Bruker, 2005)
Geometry. All esds (except the esd in the dihedral angle between two l.s. planes) are estimated using the full covariance matrix. The cell esds are taken into account individually in the estimation of esds in distances, angles and torsion angles; correlations between esds in cell parameters are only used when they are defined by crystal symmetry. An approximate (isotropic) treatment of cell esds is used for estimating esds involving l.s. planes.
Refinement. Refinement of *F*^2^ against ALL reflections. The weighted *R*-factor wR and goodness of fit *S* are based on *F*^2^, conventional *R*-factors *R* are based on *F*, with *F* set to zero for negative *F*^2^. The threshold expression of *F*^2^ > σ(*F*^2^) is used only for calculating *R*-factors(gt) etc. and is not relevant to the choice of reflections for refinement. *R*-factors based on *F*^2^ are statistically about twice as large as those based on *F*, and *R*- factors based on ALL data will be even larger.

### Fractional atomic coordinates and isotropic or equivalent isotropic displacement parameters (Å^2^)

**Table:**

	*x*	*y*	*z*	*U*_iso_*/*U*_eq_	
C1	0.5490 (8)	0.6188 (2)	0.9551 (8)	0.0201 (12)	
H1A	0.6135	0.6159	0.8480	0.024*	
H1B	0.4429	0.6401	0.9326	0.024*	
C2	0.5016 (8)	0.5612 (2)	1.0147 (8)	0.0201 (12)	
H2	0.4256	0.5654	1.1154	0.024*	
C3	0.3984 (9)	0.5318 (3)	0.8783 (8)	0.0256 (13)	
H3A	0.4683	0.5293	0.7744	0.031*	
H3B	0.2948	0.5540	0.8521	0.031*	
C4	0.6555 (8)	0.5265 (3)	1.0676 (8)	0.0239 (13)	
H4A	0.7150	0.5451	1.1623	0.029*	
H4B	0.7369	0.5238	0.9723	0.029*	
N1	0.6553 (7)	0.6496 (2)	1.0812 (7)	0.0213 (11)	
H1C	0.6816	0.6838	1.0400	0.032*	
H1D	0.7537	0.6303	1.1013	0.032*	
H1E	0.5954	0.6532	1.1789	0.032*	
Cl1	0.05791 (19)	0.63163 (6)	1.02923 (19)	0.0229 (3)	
Cl2	−0.1125 (3)	0.7500	1.2974 (2)	0.0194 (4)	
Cl3	0.2609 (3)	0.7500	0.6719 (3)	0.0238 (4)	
Pb1	0.08434 (4)	0.7500	0.99093 (4)	0.01374 (9)	

### Atomic displacement parameters (Å^2^)

**Table:**

	*U*^11^	*U*^22^	*U*^33^	*U*^12^	*U*^13^	*U*^23^
C1	0.019 (3)	0.018 (3)	0.023 (3)	0.002 (2)	−0.002 (2)	0.001 (2)
C2	0.020 (3)	0.021 (3)	0.019 (3)	0.000 (2)	−0.003 (2)	−0.001 (3)
C3	0.031 (4)	0.020 (3)	0.026 (3)	−0.004 (3)	−0.009 (3)	0.000 (2)
C4	0.023 (3)	0.021 (3)	0.028 (3)	−0.004 (3)	−0.005 (3)	−0.001 (3)
N1	0.023 (3)	0.018 (2)	0.023 (2)	−0.003 (2)	0.002 (2)	−0.003 (2)
Cl1	0.0202 (7)	0.0229 (7)	0.0257 (7)	0.0021 (5)	0.0001 (6)	−0.0037 (6)
Cl2	0.0170 (10)	0.0221 (9)	0.0192 (9)	0.000	0.0049 (7)	0.000
Cl3	0.0222 (10)	0.0297 (11)	0.0197 (9)	0.000	0.0056 (8)	0.000
Pb1	0.01269 (14)	0.01657 (14)	0.01196 (13)	0.000	0.00002 (12)	0.000

### Geometric parameters (Å, °)

**Table:**

C1—N1	1.495 (8)	C4—H4B	0.9900
C1—C2	1.512 (8)	N1—H1C	0.9100
C1—H1A	0.9900	N1—H1D	0.9100
C1—H1B	0.9900	N1—H1E	0.9100
C2—C3	1.523 (8)	Cl1—Pb1	2.8723 (15)
C2—C4	1.521 (9)	Cl2—Pb1	2.8759 (19)
C2—H2	1.0000	Cl2—Pb1^ii^	2.9002 (19)
C3—C4^i^	1.525 (9)	Cl3—Pb1^iii^	2.834 (2)
C3—H3A	0.9900	Cl3—Pb1	2.882 (2)
C3—H3B	0.9900	Pb1—Cl3^iv^	2.834 (2)
C4—C3^i^	1.525 (9)	Pb1—Cl1^v^	2.8723 (15)
C4—H4A	0.9900	Pb1—Cl2^vi^	2.900 (2)
			
N1—C1—C2	112.4 (5)	C1—N1—H1C	109.5
N1—C1—H1A	109.1	C1—N1—H1D	109.5
C2—C1—H1A	109.1	H1C—N1—H1D	109.5
N1—C1—H1B	109.1	C1—N1—H1E	109.5
C2—C1—H1B	109.1	H1C—N1—H1E	109.5
H1A—C1—H1B	107.9	H1D—N1—H1E	109.5
C1—C2—C3	109.5 (5)	Pb1—Cl2—Pb1^ii^	157.64 (8)
C1—C2—C4	113.4 (5)	Pb1^iii^—Cl3—Pb1	145.68 (9)
C3—C2—C4	111.0 (5)	Cl3^iv^—Pb1—Cl1^v^	89.10 (3)
C1—C2—H2	107.6	Cl3^iv^—Pb1—Cl1	89.10 (3)
C3—C2—H2	107.6	Cl1^v^—Pb1—Cl1	165.31 (6)
C4—C2—H2	107.6	Cl3^iv^—Pb1—Cl2	84.87 (6)
C2—C3—C4^i^	111.8 (5)	Cl1^v^—Pb1—Cl2	82.66 (3)
C2—C3—H3A	109.2	Cl1—Pb1—Cl2	82.66 (3)
C4^i^—C3—H3A	109.2	Cl3^iv^—Pb1—Cl3	91.42 (3)
C2—C3—H3B	109.2	Cl1^v^—Pb1—Cl3	97.31 (3)
C4^i^—C3—H3B	109.2	Cl1—Pb1—Cl3	97.31 (3)
H3A—C3—H3B	107.9	Cl2—Pb1—Cl3	176.29 (6)
C2—C4—C3^i^	111.4 (5)	Cl3^iv^—Pb1—Cl2^vi^	171.75 (6)
C2—C4—H4A	109.3	Cl1^v^—Pb1—Cl2^vi^	89.84 (3)
C3^i^—C4—H4A	109.3	Cl1—Pb1—Cl2^vi^	89.84 (3)
C2—C4—H4B	109.3	Cl2—Pb1—Cl2^vi^	86.875 (17)
C3^i^—C4—H4B	109.3	Cl3—Pb1—Cl2^vi^	96.83 (6)
H4A—C4—H4B	108.0		
			
N1—C1—C2—C3	−178.3 (5)	Pb1^ii^—Cl2—Pb1—Cl1^v^	89.76 (3)
N1—C1—C2—C4	−53.7 (7)	Pb1^ii^—Cl2—Pb1—Cl1	−89.76 (3)
C1—C2—C3—C4^i^	−179.2 (5)	Pb1^ii^—Cl2—Pb1—Cl2^vi^	180.0
C4—C2—C3—C4^i^	54.9 (8)	Pb1^iii^—Cl3—Pb1—Cl3^iv^	180.0
C1—C2—C4—C3^i^	−178.3 (5)	Pb1^iii^—Cl3—Pb1—Cl1^v^	90.72 (3)
C3—C2—C4—C3^i^	−54.6 (8)	Pb1^iii^—Cl3—Pb1—Cl1	−90.72 (3)
Pb1^ii^—Cl2—Pb1—Cl3^iv^	0.0	Pb1^iii^—Cl3—Pb1—Cl2^vi^	0.0

Symmetry codes: (i) −*x*+1, −*y*+1, −*z*+2; (ii) *x*−1/2, *y*, −*z*+5/2; (iii) *x*+1/2, *y*, −*z*+3/2; (iv) *x*−1/2, *y*, −*z*+3/2; (v) *x*, −*y*+3/2, *z*; (vi) *x*+1/2, *y*, −*z*+5/2.

### Hydrogen-bond geometry (Å, °)

**Table:**

*D*—H···*A*	*D*—H	H···*A*	*D*···*A*	*D*—H···*A*
N1—H1C···Cl3^iii^	0.91	2.40	3.249 (5)	156
N1—H1D···Cl1^vii^	0.91	2.44	3.196 (6)	141
N1—H1E···Cl1^vi^	0.91	2.39	3.212 (5)	150
N1—H1E···Cl2^vi^	0.91	2.84	3.337 (5)	115

Symmetry codes: (iii) *x*+1/2, *y*, −*z*+3/2; (vii) *x*+1, *y*, *z*; (vi) *x*+1/2, *y*, −*z*+5/2.
